# Automated characterisation of cerebral microbleeds using their size and spatial distribution on brain MRI

**DOI:** 10.1186/s41747-024-00544-z

**Published:** 2025-01-13

**Authors:** Vaanathi Sundaresan, Giovanna Zamboni, Robert A. Dineen, Dorothee P. Auer, Stamatios N. Sotiropoulos, Nikola Sprigg, Mark Jenkinson, Ludovica Griffanti

**Affiliations:** 1 Department of Computational and Data Sciences, Indian Institute of Science, Bengaluru, 560012 Karnataka India; 2 Nuffield Department of Clinical Neurosciences, University of Oxford, Oxford, OX3 9DU UK; 3 Dipartimento di Scienze Biomediche, Metaboliche e Neuroscienze, Universit di Modena e Reggio Emilia, Modena, 41121 Italy; 4 National Institute for Health and Care Research (NIHR) Nottingham Biomedical Research Centre, Queens Medical Centre, Sir Peter Mansfield Imaging Centre, University of Nottingham, Nottingham, NG7 2RD UK; 5 Radiological Sciences, Mental Health and Clinical Neurosciences, School of Medicine, University of Nottingham, Nottingham, NG7 2RD UK; 6 Wellcome Centre for Integrative Neuroimaging, University of Oxford, Oxford, OX3 9DU UK; 7 South Australian Health and Medical Research Institute (SAHMRI), Australian Institute for Machine Learning, School of Computer and Mathematical Sciences, University of Adelaide, Adelaide, SA 5005 Australia; 8 Department of Psychiatry, University of Oxford, Oxford, OX3 7JX UK

**Keywords:** Brain, Cerebral haemorrhage, Cerebrovascular disorders, Hemosiderin, Magnetic resonance imaging

## Abstract

**Abstract:**

Cerebral microbleeds (CMBs) are small, hypointense hemosiderin deposits in the brain measuring 2–10 mm in diameter. As one of the important biomarkers of small vessel disease, they have been associated with various neurodegenerative and cerebrovascular diseases. Hence, automated detection, and subsequent extraction of clinically useful metrics (*e.g*., size and spatial distribution) from CMBs are essential for investigating their clinical impact, especially in large-scale studies. While some work has been done for CMB segmentation, extraction of clinically relevant information is not yet explored. Herein, we propose the first automated method to characterise CMBs using their size and spatial distribution, *i.e*., CMB count in three regions (and their substructures) used in Microbleed Anatomical Rating Scale (MARS): infratentorial, deep, and lobar. Our method uses structural atlases of the brain for determining individual regions. On an intracerebral haemorrhage study dataset, we achieved a mean absolute error of 2.5 mm for size estimation and an overall accuracy > 90% for automated rating. The code and the atlas of MARS regions in Montreal Neurological Institute—MNI space are publicly available.

**Relevance statement:**

Our method to automatically characterise cerebral microbleeds (size and location) showed a mean absolute error of 2.5 mm for size estimation and an over 90% accuracy for rating of infratentorial, deep and lobar regions. This is a promising approach to automatically provide clinically relevant cerebral microbleeds metrics.

**Key Points:**

- We present a method to automatically characterise cerebral microbleeds according to size and location.
- The method achieved a mean absolute error of 2.5 mm for size estimation.
- Automated rating for infratentorial, deep, and lobar regions achieved an over 90% overall accuracy.
- We made the code and atlas of Microbleed Anatomical Rating Scale regions publicly available.

**Graphical Abstract:**

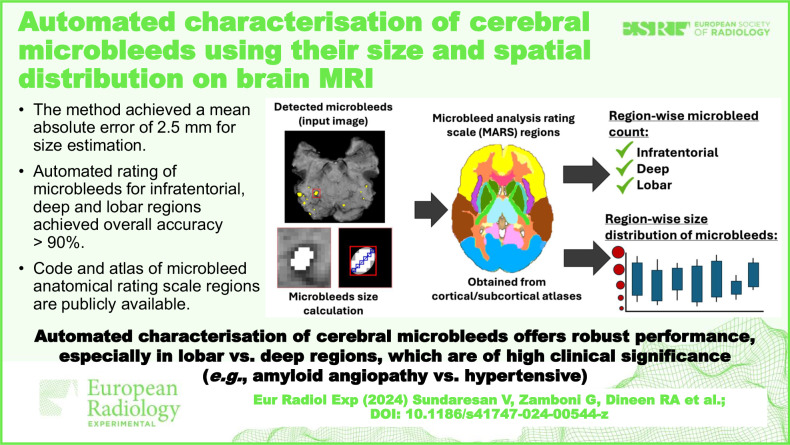

## Background

Cerebral microbleeds (CMBs) are hemosiderin deposits in the brain, mainly associated with cerebrovascular disease, Alzheimer’s disease, and cerebral amyloid angiopathy [1]. They appear as small, circular well-defined hypointense lesions of 2–10 mm in diameter on susceptibility-weighted images (SWIs). Automated CMB detection algorithms typically generate binary lesion maps of CMBs. For instance, our deep learning-based automated CMB detection method [2], detects the CMB lesions and provides the results as a binary map. However, for these methods to be translated into clinically deployable tools, they need to provide clinically useful metrics such as lesion count, size, and spatial distribution, to support the interpretation of CMB maps [3, 4].

In clinical settings, CMBs are often reported using rating scales, based on lesion count and distribution. Among the rating scales available [4, 5], the Microbleed Anatomical Rating Scale (MARS) provides CMB counts in individual lobes [4]. In the present work, as the next step of our CMB detection method [2], we propose the first automated method to characterise CMBs from their binary maps by determining their sizes and their spatial distribution in the regions used in the MARS scale. We also show that obtaining the spatial distribution of CMBs could be used to better analyse the performance of a CMB detection algorithm by providing statistics at a region-level. In future work, these metrics can be used to better understand the clinical impact of CMBs in population-level analyses.

## Methods

### Datasets and preprocessing

We used a subset of magnetic resonance imaging (MRI) data part of the Tranexamic acid for intracerebral haemorrhage−TICH-2 trial [6, 7], consisting of 50 SWI studies acquired using the standard manufacturer-specific SWI protocol from the manufacturer’s protocol tree (GE Healthcare, SWAN; Philips Healthcare, VEN_BOLD; Siemens Healthineers, SWI). For demographic details and MRI acquisition parameters, refer to [6, 7]. Out of 50 subjects, 25 subjects had CMBs. Manual segmentations and MARS ratings for CMBs were available for all 25 subjects with CMBs. CMBs were 185 in total, mean ± standard deviation 7.5 ± 12.6; median 5. For evaluation purposes, we included in our experiments all CMBs that were labelled as either ‘definite’ or ‘possible’ in the manual annotations as specified in the MARS rating form, according to the definitions described by Gregoire et al [4]. Also, note that CMBs belonging to both the above categories were used for training our segmentation method [2].

As a first step, we reoriented the images to match the orientation of standard Montreal Neurological Institute (MNI) space using the Oxford Centre Functional Magnetic Resonance Imaging of the Brain (FMRIB) software library (FSL) tool fslreorient2std^1^ and skull stripped them using FSL Brain Extraction Tool [8], followed by bias field correction using FSL FMRIB’s Automated Segmentation Tool−FAST [9]. For getting binary maps for CMB detection, we used our CMB segmentation method proposed by Sundaresan et al [2]. In the method, we detected initial candidates using a shallow 3-layer U-Net [10]. Later, these candidates were discriminated into true CMBs and mimics using a student model trained using a distillation framework, followed by a morphological clean-up step to reduce false positives.

### Automated characterisation of CMBs

**Fig. 1:**
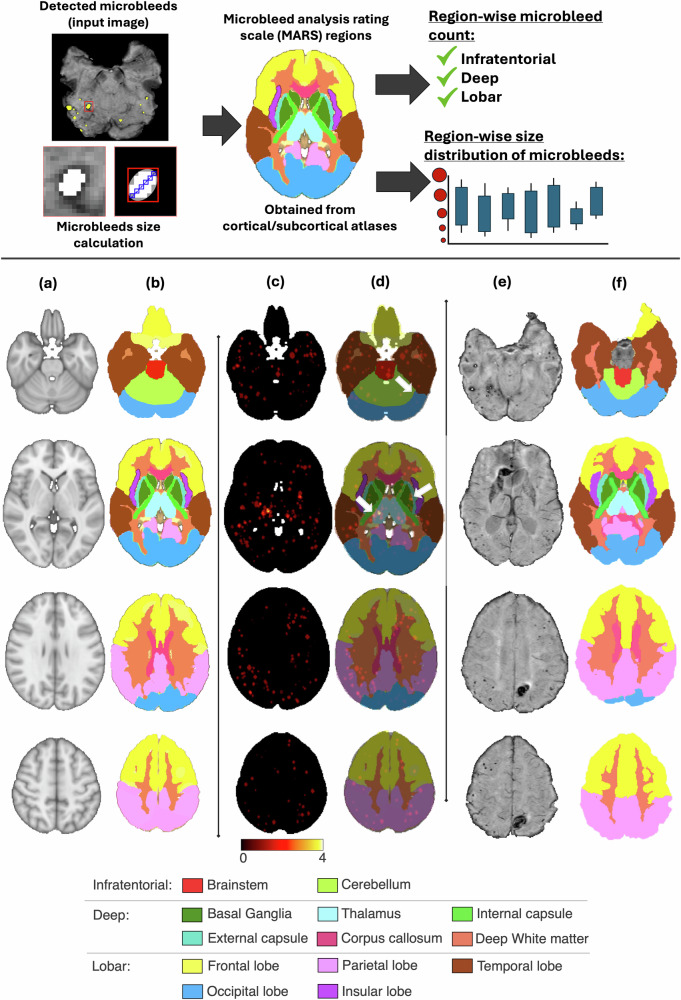
Region-wise characterisation of CMBs and MARS rating regions from the structural atlases. Top panel: Determination of CMB sizes and their spatial distribution. For major axis length estimation, the magnified patches show the CMB mask overlayed on the input image and the longest diameter voxels (blue) within the bounding box (red). For instance, a CMB with an axis length of 6 voxels and 0.9 mm/voxel resolution, has a diameter of 5.4 mm. Bottom panel: (**a**) MNI T1-weighted structural image, (**b**) regions for MARS rating in the MNI space, (**c**) CMB prevalence map (number of subjects with CMBs in each voxel) in the MNI space (for visualisation purposes only), (**d**) prevalence map with MARS regions overlaid (thus indicating structure-wise CMB distribution), and arrows showing examples of potential misclassifications, (**e**) example susceptibility-weighted image in the subject space, (**f**) MARS regions formed by registering atlases to the subject space. Legend for various structures is shown at the bottom. CMBs, Cerebral microbleeds; MARS, Microbleed Anatomical Rating Scale; MNI, Montreal Neurological Institute

The aim is to obtain the size of CMBs from the binary maps and use structural brain atlases to define the regions for obtaining region-wise counts of CMBs (Fig. 1).

#### CMB size

Within the binary map of CMBs, we considered a bounding box around each CMB. We determined the longest diameter (in voxels) of the ellipsoid that would fit the bounding box. The criterion is that the ellipsoid should have the same second-order moment as the CMB (*i.e*., with the same spread and orientation). For each CMB, the length of the major axis in voxels was scaled with voxel dimensions (mm/voxel) to obtain the final diameter in mm.

#### Spatial distribution of CMBs

As a first step, we extracted the regions (*e.g*., infratentorial) and structures within regions (*e.g*., brainstem) used in the MARS scale from MRI atlases. Table 1 reports the structures considered and the thresholds used to binarise the probabilistic atlases. We later registered the structures from the MNI space to the subject space using linear registration with 12 degrees of freedom with FSL Linear Image Registration Tool−FLIRT [11] and thresholded them at 0.5 to obtain binary masks in the single-subject space. We performed a morphological dilation operation (using a structuring element with the size of 3 voxels, empirically chosen from a tested range of 2, 3, 5, 7, and 9 voxels) on the cortical lobes mask. This increased the extent of the cortex to include the grey-white matter junctions as ‘lobar’ region. Also, deep structures such as internal/external capsules and corpus callosum were excluded from the white matter mask since they were considered separately. The Python code and MARS region atlas are available at https://github.com/v-sundaresan/microbleed-size-counting.

**Table 1:** MARS rating scale regions and structures for CMBs, along with statistics of subject-wise counts of detected CMBs (including true and false positives) for each structure

MARS scale		Atlases	Proportion of subjects with at least one detected CMB (%)	Maximum number of subject-wise count of detected CMBs
Region	Structure			
Infratentorial	Brainstem	Harvard-Oxford subcortical structural [13, 14] (threshold 50)	3.7	2
	Cerebellum	MNI structural [15, 16]	55.6	4
Deep	Basal ganglia	Harvard-Oxford subcortical structural [13, 14] (threshold 50)	20.0	1
	Thalamus		14.2	2
	Internal capsule	JHU ICBM-DTI-81 white matter labels [17–19]	3.7	1
	External capsule		24.8	4
	Corpus callosum		3.7	1
	Deep white matter	Harvard-Oxford subcortical structural [13, 14] (threshold 60)	37.1	6
Lobar	Frontal	MNI structural [15, 16]	100.0	11
	Parietal		100.0	14
	Temporal		81.2	8
	Occipital		100.0	17
	Insular		85.2	11

Atlases used for the individual structures are reported. In case a probabilistic atlas was used, the threshold applied is reported in parentheses. References are reported in square brackets. Basal ganglia include caudate plus lentiform nuclei

*CMB* Cerebral microbleeds, *DTI* Diffusion tensor imaging, *ICBM* International Consortium of Brain Mapping, *JHU* Johns Hopkins University, *MNI* Montreal Neurological Institute

Secondly, we counted the number of CMBs within each structure/region, to derive the MARS rating. Figure 1 shows sample regions from the structural atlases, the ‘CMB prevalence map’ (heatmaps of CMBs) with the atlas overlaid in the MNI space, and the atlas regions in an individual subject space. For each CMB, we determined its centroid as an average of the CMB lesion coordinates in *x*-, *y*- and *z*-dimensions*.* We then obtained the count of CMB centroids to obtain the structure-wise count of CMBs and aggregated the number of CMBs to provide region-wise CMB count.

### Experimental setup and performance metrics

For CMB size estimation, we determined the absolute error between the sizes of the detected CMBs and those of the manually segmented CMBs. For the evaluation of automated MARS ratings, we compared the region-wise CMB counts from the CMBs that were correctly predicted by our segmentation algorithm [2] (true positives) to the manually rated MARS subscores. For region-wise count of CMBs, we used region-wise sensitivity, region-wise precision, and region-wise F1 measure with respect to the manual ratings.

## Results

**Fig. 2:**
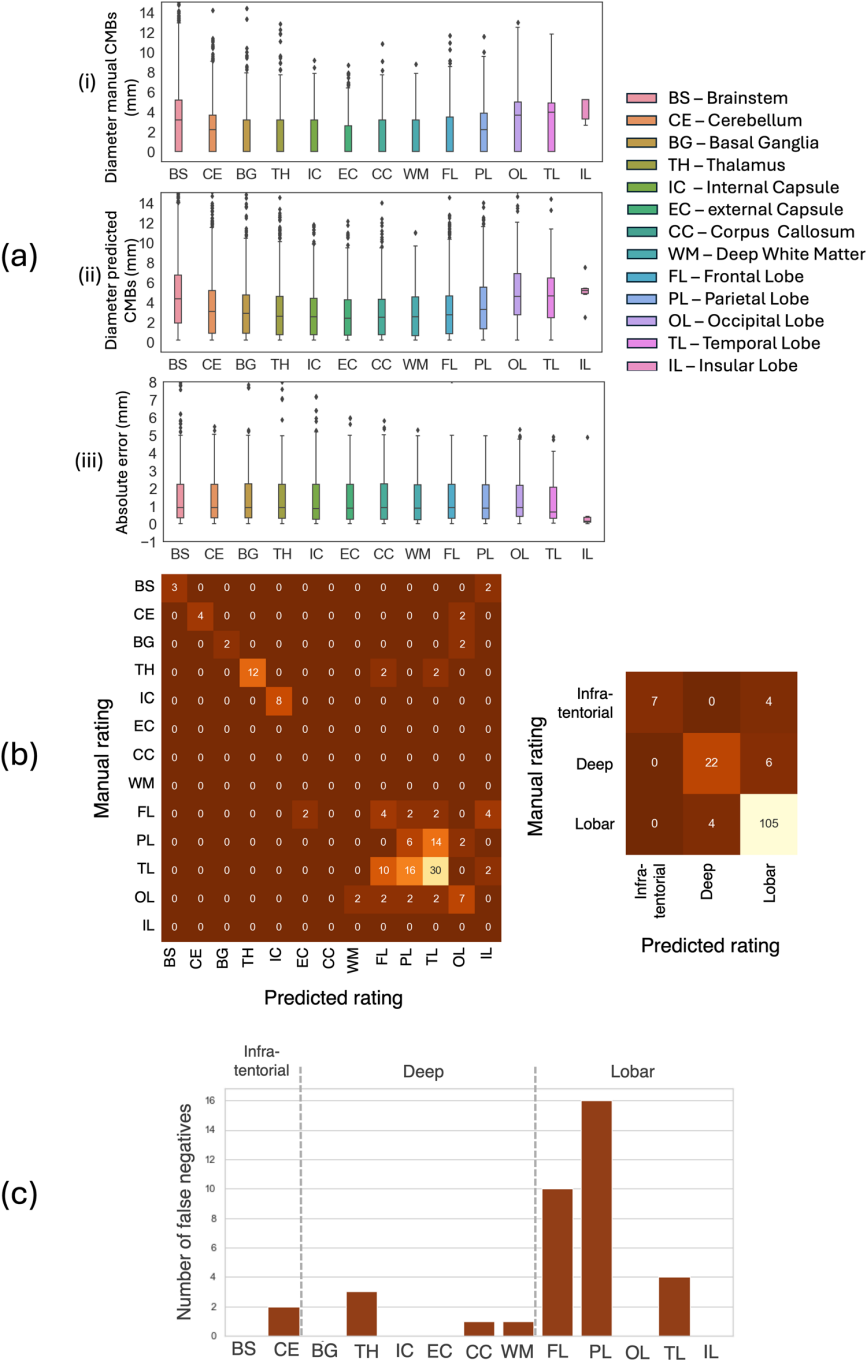
CMB size estimation and rating results on images from the Tranexamic acid for hyperacute primary intracerebral haemorrhage−TICH-2 trial. **a** Structure-wise distribution of CMB sizes is shown for (i) manually annotated CMBs, (ii) predicted CMBs, and (iii) absolute error between manually annotated and predicted CMBs. **b** Confusion matrices for automated CMB rating for individual structures and regions using detected true positive CMBs. **c** Bar plots showing the number of false negatives for individual structures. CMBs, Cerebral microbleeds

We show in Fig. 2a the boxplots of structure-wise sizes of manually segmented and predicted CMBs, along with the absolute error between them. The error values mainly ranged between 1 and 2 mm, thus resulting in very similar median values in the boxplots, except for the insular lobe, where only a few CMBs were present. We also observed that around 35% of automatically detected CMBs differed from the manually segmented CMBs by less than 1.0 mm, and only 11 of 185 CMBs (6%) had a difference larger than 6.0 mm. We obtained a mean absolute error value of 2.5 mm between the manually segmented and predicted CMBs. Figure 2b shows the confusion matrices (both region- and structure-wise) between the manual and automated ratings obtained for predicted CMBs. Our automated MARS rating method achieved a region-wise sensitivity of 0.64, 0.79, 0.96, a region-wise precision of 1.00, 0.85, 0.91, and a region-wise F1-measure of 0.78, 0.82, 0.93 for infratentorial, deep and lobar regions respectively, with an overall accuracy of 91%. Figure 2c shows the structure-wise distribution of CMBs that were not detected by our segmentation method (false negatives). From the figure, it is evident that most of the false negatives occurred in the parietal and frontal lobes. Table 1 reports the statistics of the subject-wise predicted CMB counts across all subjects.

## Discussion

In this work, we extracted the size (diameter) of CMBs and automatically calculated MARS scores. We observed good agreement between the diameters of manually segmented and predicted CMBs. Most of the cases with high absolute error (≈ 6 mm) were due to small fragments of vessels that were segmented along with the CMBs (see Sundaresan et al [2] for example cases).

The MARS regions obtained using our method align well with those of clinically provided manual rating (indicated by a high performance of 91% overall accuracy). While the results reported in Table 1 include all CMBs identified by the segmentation algorithm, we only considered true positive CMBs when determining the accuracy of the automated MARS ratings (as there are no manual MARS ratings for false detections). The misclassifications in MARS scores occurred mainly at the structure-level, near the boundaries of structures in the MARS atlas (Fig. 1d, bottom panel). This could be mainly due to: (i) errors occurring during registration of the MNI atlas to subject space (non-linear registration was also attempted, but was not satisfactory due to poor contrast of subcortical regions in the white matter); and (ii) the size of kernels used for dilating atlas structures (the chosen size was a tradeoff between filling gaps between structures and maintaining correct CMB count across regions).

Additionally, misclassifications could also be due to outline errors from the under-/over-segmentation of predicted CMBs, especially in the region/structure boundaries, hence shifting the centroids to the adjacent regions. The performance measures obtained for the three main regions (infratentorial, deep, lobar) were highly robust, despite minor misclassifications across the individual structures (*e.g*., basal ganglia) (Fig. 2). Clinically, CMBs are studied often at the region-level (*e.g*., lobar CMBs are associated with cerebral amyloid angiopathy and Alzheimer’s disease, while deep CMBs are associated with cerebrovascular diseases [12]).

Given a high region-wise accuracy for CMB counting and accurate size determination, the proposed method could be useful in extracting imaging-based CMB biomarkers starting from binary CMB maps obtained either with the same algorithm used in this work [2] or others [3].

The results also gave insights into the performance of our CMB segmentation algorithm [2]. We observed more false positives in the lobar regions, indicating that their possible source could be the edge artefacts close to the skull and the troughs of the cortical sulci. False negatives were mostly the subtle and small CMBs, especially when they occur closer to the blood vessels. Overall, the count of CMBs in individual regions can be used as location-based soft priors/weights for improving the accuracy of automated CMB detection methods.

In conclusion, we proposed an automated characterisation of CMBs based on their size and spatial distribution using the MARS rating scale. Our method was affected by a mean absolute error of 2.5 mm between the diameters of manually segmented and predicted CMBs. For automated rating of CMB counts, we obtained an over 90% overall accuracy for predicted CMBs, indicating the reliability of the atlas registration performed in our method. A future direction of this work will be to investigate, in larger datasets, the relationship between the region-wise distribution of CMBs and various clinical/demographic factors to further assess the impact of CMBs at the population level.

## Data Availability

The TICH-2 MRI sub-study data can be shared with bona fide researchers and research groups on written request to the sub-study PI RD (ku.ca.mahgnitton@neenid.bor). Proposals will be assessed by the PI (with advice from the TICH-2 trial Steering Committee if required) and a Data Transfer Agreement will be established before any data are shared.

## Abbreviations

CMBs: Cerebral microbleeds
FMRIB: Functional Magnetic Resonance Imaging of the Brain (Oxford Centre)
FSL: FMRIB software library
MARS: Microbleed Anatomical Rating Scale
MNI: Montreal Neurological Institute
MRI: Magnetic resonance imaging
SWI: Susceptibility-weighted imaging

## Footnotes

1 https://fsl.fmrib.ox.ac.uk/fsl/docs/#/utilities/fslutils?id=fslreorient2std

## Code availability

The Python code for the implementation of the method and the atlas of MARS regions in the MNI space are available at https://github.com/v-sundaresan/microbleed-size-counting.
